# SIRT5 is important for bacterial infection by regulating insulin secretion and glucose homeostasis

**DOI:** 10.1007/s13238-020-00709-7

**Published:** 2020-05-15

**Authors:** Cuiping Zhang, Ke Wang, Zuojian Hu, Lujie Yang, Bin Wei, Shan Li, Xue Qin, Pengyuan Yang, Hongxiu Yu

**Affiliations:** 1 Minhang Hospital & Institutes of Biomedical Sciences, Department of Systems Biology for Medicine, School of Basic Medical Sciences, Fudan University, Shanghai, 200032 China; 2 Department of Clinical Laboratory, First Affiliated Hospital of Guangxi Medical University, Nanning, 530021 China; 3 School of Life Science, Shanghai University, Shanghai, 200444 China; 4 Minhang Hospital & Institutes of Biomedical Sciences, Department of Systems Biology for Medicine, School of Basic Medical Sciences, Fudan University, Room 501, Mingdao Building, 131 Dongan Road, Shanghai, 200032 China; 5 Minhang Hospital & Institutes of Biomedical Sciences, Department of Systems Biology for Medicine, School of Basic Medical Sciences, Fudan University, Room 201, Mingdao Building, 131 Dongan Road, Shanghai, 200032 China

**Dear Editor,**

Recent studies indicate that cellular metabolism plays a key role in supporting immune cell development, maintenance and function (Norata et al., 2015). For example, sensing of microbial ligands by macrophages triggers increased glucose metabolism, which delivers energy to support antimicrobial inflammation and the production of cytokines. This effect further enhances the generation of mitochondrial reactive oxygen species (ROS) to fight infections. Tucey et al. show that maintaining host glucose homeostasis is important to prevent life-threatening fungal infection (Tucey et al., 2018). This phenomenon emerges as a bedrock of the emerging concept of how metabolism and immunity are integrated based on bioenergy requirements.

Sirtuins contribute to dynamic shifts in immunity, metabolism, and bioenergetics during inflammation (Vachharajani et al., 2016). Therefore, targeting Sirtuins is likely to deliver new opportunities for manipulating immunometabolism as an anti-inflammatory strategy. However, better understanding of Sirtuin biology and its role in regulation of inflammation is in its infancy. SIRT1 acts as a key control point for regulating metabolism and inflammatory outputs from T cells (Liu et al., 2012). In our previous work, we demonstrated that SIRT5 plays an important role in preventing dextran sulfate sodium (DSS)-induced colitis by regulating glycolysis in macrophages (Wang et al., 2017). Inflammation in DSS-induced colitis is not of infectious origin. We would like to know the function of SIRT5 in infection-induced inflammation and host defense.

To study the role of SIRT5 in infection response, we treated littermate wild-type (WT) and *Sirt5*-deficient (*Sirt5*^−/−^) mice with lipopolysaccharide (LPS). When *Sirt5*^−/−^ mice were treated with LPS for 3 h, the level of blood glucose decreased and the level of serum insulin increased significantly (Fig. 1A and 1B), accompanied with increased concentration of circulating IL-1β (Fig. 1C). Peritoneal macrophages (PMs) from *Sirt5*^−/−^ mice treated with LPS also expressed higher level of *Il-1β* mRNA and secreted more IL-1β (Fig. S1A and S1B). These results indicated that SIRT5 has a potential role in regulating glucose homeostasis and *Sirt5* deficiency boosts IL-1β production in inflammatory response.

**Figure 1:**
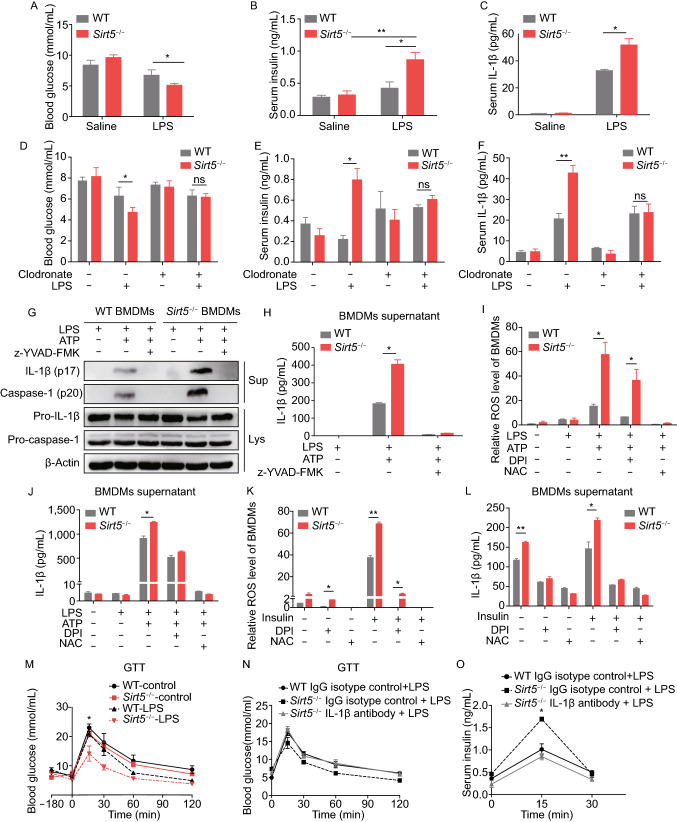
**Macrophage-derived IL-1β stimulates insulin in** ***Sirt5***^**−/−**^ **mice, and both synergistically regulate glucose homeostasis**. (A) Blood glucose, (B) serum insulin and (C) circulating IL-1β were determined before and after intraperitoneal injection of LPS for 3 h (1 mg/kg) into wild-type (WT) mice and *Sirt5*^−/−^ mice (*n* = 3–7/group). (D) Blood glucose level, (E) serum insulin and (F) circulating IL-1β were determined before and after LPS stimulation upon intraperitoneal injection of 10 mL/kg clodronate or PBS liposomes (*n* = 3/group). (G) Immunoblotting for IL-1β (p17), activated caspase-1 (p20) in supernatant (Sup), and pro-IL-1β, pro-caspase-1, β-actin in cell lysates (Lys) in the absence or presence of the caspase-1 inhibitor z-YVAD-FMK (10 µmol/L, 1 h). (H) ELISA for IL-1β in supernatant from LPS-primed BMDMs (100 ng/mL, 5.5 h) in the absence or presence of the caspase-1 inhibitor z-YVAD-FMK (10 µmol/L, 1 h). (I) ROS and (J) IL-1β production were determined in resting or LPS-primed BMDMs stimulated with or without ROS inhibitors N-acetyl-cysteine (NAC, 25 mmol/L, 1 h) or NAPDH oxidase inhibitor diphenyleneiodonium (DPI, 25 μmol/L,1 h). (K) ROS and (L) IL-1β production in LPS-primed BMDMs stimulated with or without insulin (1 μg/mL, 5 min), NAC (25 mmol/L, 1 h) or DPI (25 μmol/L, 1 h). (M) Concentration of blood glucose during an intraperitoneal GTT in WT or *Sirt5*^−/−^ mice after injection of saline (control) or LPS (1 mg/kg) for 180 min (*n* = 3/group); 0 min indicates GTT start time throughout GTT analyses. (N) Concentration of blood glucose, and (O) circulating insulin (G) during an intraperitoneal GTT in mice 180 min after injection of LPS (1 mg/kg) with IgG isotype control (100 μg each) or IL-1β neutralizing antibody (100 μg each) (*n* = 3/group). Data represent mean ± SEM, *P < 0.05, **P < 0.01

Then, we wanted to know the source of the elevated IL-1β. The gating strategies of flow cytometry for immune cells have been shown in Fig. S1C. We found that the F4/80^+^/CD11b^+^ macrophages accounted for the largest proportion of the immune cells isolated from peritoneal cavity of *Sirt5*^−/−^ mice treated with LPS (Fig. S1D), which is consistent with a previous interesting report (Dror et al., 2017). Depletion of the peritoneal macrophages by intraperitoneal injection of clodronate liposomes (Fig. S1E) eliminated the effect induced by LPS treatment in *Sirt5*^−/−^ mice. The blood glucose, circulating insulin and IL-1β level in LPS-treated *Sirt5*^−/−^ mice showed no significant difference with that in WT mice (Fig. 1D–F). Therefore, LPS induced elevation of IL-1β in *Sirt5*^−/−^ mice is mainly from the intraperitoneal macrophages.

The NLRP3 inflammasome was shown to be a pivotal player in sepsis and the activation of the NLRP3 inflammasome cause IL-1β and IL-18 production (Ting et al., 2008). So, we assumed that the inflammasome activation might be enhanced by LPS treatment in *Sirt5*^−/−^ macrophages. In *Sirt5*^−/−^ bone marrow derived macrophages (BMDMs) cells, treatment with LPS did not change the level of SIRT5 protein, while significantly upregulated mRNAs level of NRLP3 and IL-1β, compared to the WT counterparts (Fig. S2A–C). With LPS priming, ATP promoted the cleavage of caspase 1 and the maturation of IL-1β. The result showed both signal 1 and signal 2 of the NLRP3 inflammasome were activated (Fig. 1G) (Hao et al., 2017). The IL-1β released into BMDMs supernatant was confirmed by ELISA (Fig. 1I). While the caspase-1 inhibitor zYVAD abolished IL-1β release in both WT and *Sirt5*^−/−^ BMDMs (Fig. 1G and 1H). Furthermore, we investigated the mechanisms involved in inflammasome activation. ROS are pivotal for inflammasome activation (Schroder and Tschopp, 2010). Treatment with LPS and ATP increased ROS production in *Sirt5*^−/−^ macrophages when compared to WT macrophages, and this was inhibited by ROS inhibitor N-acetyl-cysteine (NAC) and NAPDH oxidase inhibitor diphenyleneiodonium (DPI) (Fig. 1I). Inhibition of ROS production also suppressed IL-1β release as assessed by ELISA (Fig. 1J). Together, these findings reveal that LPS-induced IL-1β secretion in *Sirt5*^−/−^ BMDMs depended on NLRP3 inflammasome activation and ROS production.

Published *in vitro* studies (Hajmrle et al., 2016) have demonstrated that IL-1β enhance the glucose-stimulated secretion of insulin from islets. Meanwhile, resident macrophages isolated from various tissues express insulin receptor (InsR) (Dror et al., 2017). Increased InsR expression in the macrophages of diet-induced obesity (DIO) mice has been reported and insulin reversely reinforced the inflammatory state of macrophages. Although we did not find upregulation of InsR in *Sirt5*^−/−^ BMDMs than WT counterparts (data not shown), we did observe that insulin treatment significantly increased the production of IL-1β in M1 BMDMs derived from *Sirt5*^−/−^ mouse (Fig. S3A). Also, insulin treatment increased the production of ROS and IL-1β in *Sirt5*^−/−^ BMDMs primed with LPS. Both ROS and IL-1β were suppressed by NAC or DPI (Fig. 1K–L). PI3K-AKT and MAPK (JNK, p38 and ERK) signaling pathway have been shown to have a close relationship with insulin signal transduction. Then we measured these pathways. The extent of insulin-induced phosphorylation of the kinase AKT was greater in *Sirt5*^−/−^ BMDMs than WT BMDMs (Fig. S3B, lanes 4 and 8). We noticed that the single insulin treatment did not induce p-AKT (Fig. S3B, lanes 1 and 2), which may result from the type of macrophage. According to the work of Dror et al, insulin has activity in M1 macrophages but not in naive or M2 macrophages (Dror et al., 2017). Insulin also enhanced higher phosphorylation of JNK in *Sirt5*^−/−^ BMDMs than WT BMDMs (Fig. S3B, lanes 4 and 8). Together, these findings indicated that increased IL-1β concentrations enhanced insulin secretion. Secreted insulin may bind to InsR on macrophages, leading to enhanced AKT and JNK phosphorylation, ROS production, as well as IL-1β production.

We also treated WT or *Sirt5*^−/−^ mice with LPS, followed by an intraperitoneal glucose tolerance test (GTT) and insulin tolerance test (ITT). Injection of LPS improved glucose tolerance in *Sirt5*^−/−^ mice (Figs. 1M and S4A), but had no effect on insulin sensitivity (Fig. S4B and S4C). *Sirt5*^−/−^ mice treated with IL-1β neutralizing antibody exhibited an impaired glucose tolerance during GTT when compared with those receiving IgG (Figs. 1N and S4D). Also, the level of serum insulin in *Sirt5*^−/−^ mice administered by IL-1β neutralizing antibody decreased significantly during GTT, when compared with those receiving IgG (Fig. 1O). Specific immunostaining of mouse pancreatic tissue sections with insulin antibody confirmed that blocking IL-1β can suppress insulin secretion (Fig. S4E). In order to investigate the pathways mediating insulin secretion in islet β-cell, we further isolated islets from WT or *Sirt5*^−/−^ mice and evaluated the *ex vivo* impact of SIRT5 on insulin secretion. *Sirt5*^−/−^ led to enhanced AKT phosphorylation in islets (Fig. S4F). IL-1β or LPS-stimulated insulin secretion was markedly increased in *Sirt5*^−/−^ islets (Fig. S4G and S4H). Both basal and glucose-stimulated insulin secretion were significantly increased in *Sirt5*^*−/−*^ islets (Fig. S4I). These results indicated that during the inflammatory response, *Sirt5* deficiency leads to IL-1β production and insulin secretion, which regulates glucose homeostasis. There was a positive feedback between macrophage and β cell. Previous finding also showed that SIRT6 was essential for proper glucose homeostasis (Zhong et al., 2010).

To better understand the role of macrophage SIRT5 in regulating glucose homeostasis, we deleted *Sirt5* in macrophages by transplanting WT recipient mice with WT (WT→WT) or *Sirt5*^−/−^ (*Sirt5*^−/−^→WT) bone marrow cell. Upon reconstitution, *Sirt5*^−/−^→WT chimeras displayed decreased level of blood glucose, increased level of serum insulin and IL-1β, compared with those of WT→WT (Fig. S5A–C). The assay of GTT and ITT showed same results with Fig.1M and Fig.S4B, although it did not reach statistical significance (Fig. S5D and S5E).

In order to characterize the effect of SIRT5 on glucose homeostasis in infection, we challenged the mice with *Salmonella typhimurium* (strain SL1344). The blood glucose of WT and *Sirt5*^−/−^ mice with *S*. *typhimurium* show a trend of decline during the infection; when compared to WT mice, *Sirt5*^−/−^ mice had a lower level of blood glucose at 12 h post bacterial infection (Fig. 2A). Accordingly, *Sirt5*^−/−^ mice had significantly higher level of serum insulin at 12 h and 24 h post infection than WT mice (Fig. 2B). The level of IL-1β in serum and supernatant of PMs from infected *Sirt5*^−/−^ mice increased much more than WT mice as expected (Figs. 2C and S6A). Also, *Il-1β* mRNA expression increased in PMs, liver and spleen at 24 h post infection and the increase was higher in *Sirt5*^−/−^ mice than WT mice (Fig. S6B–D). In order to measure bacterial dissemination, mice were sacrificed at 24 h post infection with *S*. *typhimurium* i.p.; livers and spleens were extracted and the bacterial load determined by colony-forming units (CFUs)/mg. The bacterial load of livers and spleens from infected *Sirt5*^−/−^ mice were much higher than that in WT mice (Fig. 2D). Together, these data demonstrate that SIRT5 is important in maintaining glucose homeostasis for controlling inflammation during bacterial challenge.

**Figure 2:**
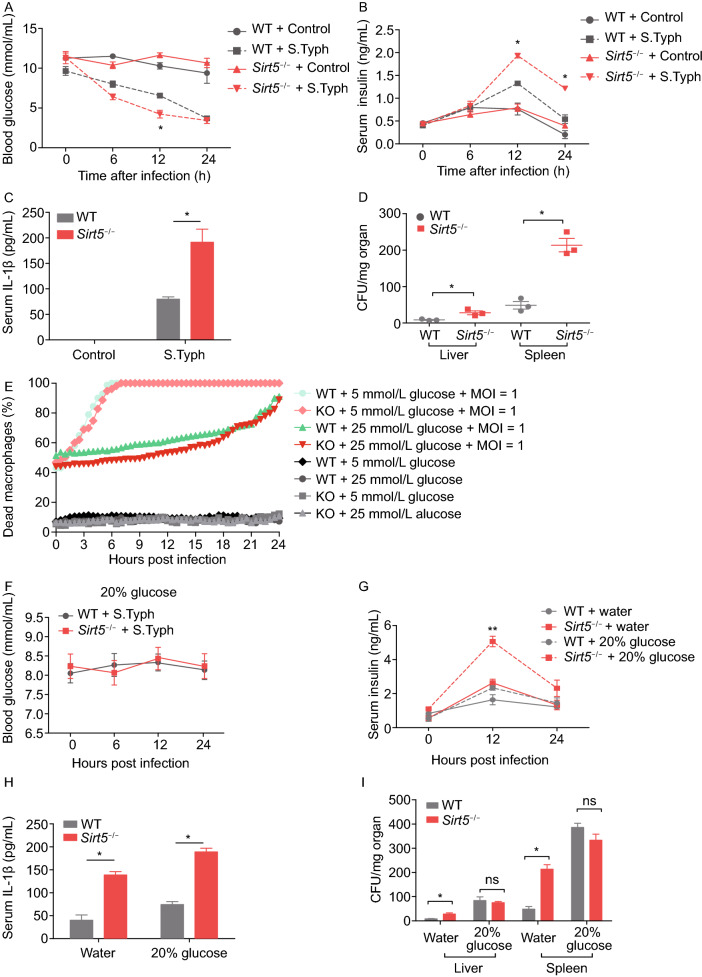
***Sirt5*** **deficiency promotes bacterial dissemination in** ***Salmonella typhimurium*** **infection model, and glucose supplementation improves host inflammatory response**. (A and B) Concentration of blood glucose (A) and insulin (B) after a single intraperitoneal injection of *S*. *typhimurium* SL1344 strain (1 × 10^6^ CFU/mouse) for 6 h, 12 h and 24 h. (C) Mice were infected as in (A), and sacrificed 24 h post infection. IL-1β in serum was measured by ELISA. (D) Livers and spleens were extracted after 24 h infection and the CFU/mg organ were determined (*n* = 3/group). (E) Comparison of WT and *Sirt5*^−/−^ macrophage cell death in medium containing different concentrations of glucose. *S*. *typhimurium* was used to infect macrophages at 1 MOI. (F) Blood glucose levels over time in *S*. *typhimurium* infected mice fed with 20% glucose in the drinking water (1 × 10^6^ CFU/mouse i.p., *n* = 3/group). (G) Concentration of serum insulin over time in *S. typhimurium* infected mice fed with drinking water or 20% glucose in the drinking water (1 × 10^6^ CFU/mouse i.p., *n* = 3/group). (H) Mice were infected as in (F), and sacrificed 24 h post infection. IL-1β in serum was measured by ELISA. (I) Livers and spleens were extracted after 24 h infection and the CFU/mg organ were determined (*n* = 3/group). Data represent mean ± SEM, *P < 0.05, **P < 0.01

We next asked if maintaining glucose homeostasis in *Sirt5*^−/−^ mice can improve the outcomes of bacterial infection. *In vitro* analysis showed that supplementation with high glucose (25 mmol/L) medium delayed macrophage cell death in both WT and *Sirt5*^−/−^ BMDMs infected with bacteria (Fig. 2E). Bacteria seem to preferentially use glucose compared with fungi (Tucey et al., 2018). Upon glucose supplementation, bacteria proliferate very rapidly. The media in the *in vitro* experiment presented in Fig. 2E became yellow and turbid very fast, which might interfere with our data, but we can still observe the BMDMs cell death in *Sirt5*^−/−^ were slightly decreased from 0 h to 18 h when compared with the WT counterparts.

This implies the glucose is essential for macrophage to mount effective anti-bacterial response. In an *in vivo* study, the blood glucose of mice fed with 20% glucose maintained higher levels of glucose during the infection (Fig. 2F). The serum insulin in infected *Sirt5*^−/−^ mice fed with glucose was significantly increased (Fig. 2G). We observed that serum levels of IL-1β and supernatant from PMs were much higher after glucose feeding, especially in *Sirt5*^−/−^ mice (Figs. 2H and S6E). *Il-1β* mRNA expression increased in PMs, liver and spleen in glucose-fed *Sirt5*^−/−^ mice (Fig. S6F–H). When the mice were fed with drinking water, the liver and spleen bacterial burden were higher in *Sirt5*^−/−^ mice than that in WT mice (Fig. 2D). We observed that glucose-feeding significantly increased liver and spleen bacterial burden in both *Sirt5*^−/−^ and WT mice, compared with the group fed drinking water, the increased portions of *Sirt5*^−/−^ group was smaller than that in WT group (Fig. 2I). This indicates that administration of glucose rescues the infection-induced hypoglycemia, and higher glucose promotes host inflammatory response against bacterial infection.

We appreciate that Heinon et al. have recently reported that *Sirt5* deficiency does not compromise the response to bacterial infection (Heinonen et al., 2018). We used different background mice and bacterial strains from them. Maybe this is where the different results come from.

Accumulated evidence have shown that the IL-1β is a inducer of both insulin resistance and impaired pancreatic islet function (Wen et al., 2011). Despite this, findings suggest that the actions of IL-1β on glycemic control may be pleiotropic in nature, with IL-1β signaling exerting both positive and negative effects *in vivo*. For example, examining of IL-1β receptor deficiency or antagonism in *in vivo* animal models, as well as in clinical studies of type 2 diabetic (T2D) patients indicate that the positive actions of IL-1β on glycemic control (Hajmrle et al., 2016). In line with this, the acute postprandial rise in IL-1β production has been demonstrated to contribute to insulin secretion and is necessary for normal glycemic control (Dror et al., 2017). Our previous work highlighted the deficiency of *Sirt5* in promoting IL-1β production by regulating PKM2 (Wang et al., 2017). Our current study confirmed macrophage-derived IL-1β stimulates insulin secretion in *Sirt5*^−/−^ mice, while has no effect on insulin sensitivity. Furthermore, there is a crosstalk between macrophage and pancreatic β cells, macrophage derived IL-1β and pancreatic β cells secreted insulin synergistically regulate glucose homeostasis, which is consistent with Dror et al.’s work. The working model of this work has been shown in Fig. S7.

In addition to IL-1β, another cytokine interferon-γ (IFN-γ) has been recently reported to have a pivotal role in regulating glucose homeostasis, suggesting that immune-endocrine interactions operate at multiple levels to increase systemic insulin concentrations (Sestan et al., 2018).

In addition to pro-inflammatory cytokines influencing insulin pathway and glucose homeostasis to regulate infection, Tsai et al. recently report that InsR signaling has an impact on T cell glucose metabolism and amino acid handling (Tsai et al., 2018).

In this work, we focus on the role of SIRT5 in whole body metabolism, which is important for anti-systemic bacterial infection. This work suggests that manipulating SIRT5 is an attractive option for the treatment of infection.

## Electronic supplementary material

Below is the link to the electronic supplementary material.

Supplementary material 1 (PDF 975 kb)
