# Supplementary material for: SIRT5 is important for bacterial infection by regulating insulin secretion and glucose homeostasis
Source: Protein Cell. 2020 May 15;11(11):846–51. doi: 10.1007/s13238-020-00709-7 (PMC7647967; doi:10.1007/s13238-020-00709-7)
Supplement: Supplementary file 1 — Supplementary material 1 (PDF 975 kb) [file 13238_2020_709_MOESM1_ESM.pdf]

SIRT5 is important for bacterial infection by regulating insulin secretion and glucose homeostasis

Cuiping Zhang[1,2#], Ke Wang[1#], Zuojian Hu[2#], Lujie Yang[1], Bin Wei[3], Shan Li[2], Xue Qin[2], Pengyuan Yang[1,*], Hongxiu Yu[1,4,*]

Supplementary materials

Materials and Methods

Cell culture

BMDMs and peritoneal cells were isolated from 8-10-week-old littermate of WT or *Sirt5*$^{-/-}$ mice (mice obtained from the Jackson Laboratory). Cells were cultured in Dulbecco's Modified Eagle's Medium (DMEM) supplemented with 10% fetal bovine serum (FBS) and antibiotics (100 U/mL penicillin and 100 mg/ml streptomycin), in 5% CO_2 atmosphere at 37°C. Bone marrow from mice was differentiated into macrophages for 7 days in DMEM supplemented with 20ng/ml murine macrophage colony stimulating factor (M-CSF). To harvest the peritoneal cells, the peritoneal cavity of a mouse infused with 5 ml sterile PBS containing 1% FBS. Peritoneal macrophages from the lavage were adhered to cell culture plates for at least 6 hours in DMEM with 10%FBS, after which the supernatant and adhered cells were harvested. Macrophages were polarized into M1 or M2 phenotypes as follows: treatment with 100ng/ml LPS 5.5 h or 12 h for M1 polarization, follow by 30 min (serum-free) with 5μM ATP for IL-1β secretion into supernatant; treatment with 20ng/ml IL-4 (24 h) for M2 polarization. M1 and M2 macrophages stimulated with 1μg/ml insulin (5 min) in the presence or absence of NAC (25Mm, 1 h) or DPI (25μM,1 h). In order to inhibit caspase-1, macrophages

were treated with 10μM z-YVAD (1 h).

***In vivo* treatment administration.**

Mice were injected intraperitoneally of LPS and IL-1β neutralizing antibody at the time and dose indicated in the figure legends, followed by GTT and ITT test.

Macrophage ablation

Clodronate or PBS liposomes were injected intraperitoneally (10ml/kg body weight) 3 days before the start of the experiments. To verify macrophage depletion, mice were sacrificed after 3 days injection, and peritoneal cells were isolated as described above and macrophages were determined as $CD11b^{+}F4/80^{+}$ double-positive cells by flow cytometry.

GTT and ITT

For glucose tolerance test, mice were fasted overnight (12-16 h), then injected intraperitoneally with 2g/kg glucose. The blood glucose and insulin were measured at the time indicated in the figures. For insulin tolerance test, mice were fasted for 4-6 h starting in the morning, and injected intraperitoneally with 0.75UI/kg insulin. The blood glucose was measured at the time indicated in the figures.

***In vivo* model of *S. typhimurium* infection**

Mice were starved for 3-4 h in the morning before measurements of blood glucose levels at 0 h. Mice were injected intraperitoneally with $1x10^7$ CFU *S. typhimurium* and fed with drinking water with or without 20% glucose, then blood glucose and circulating insulin were measured at the time indicated in the figure legends. At 24 h postinfection, serum was isolated from whole blood, left to coagulate at room

temperature, and spun at 3000 rpm for 10 min at 4 ºC. PMs, liver and spleen were harvested from infected mice. The organs were homogenized in 2ml PBS, then CFU/mg liver or spleen was determined to measure bacterial dissemination.

Intracellular ROS detection

Intracellular ROS production was measured by using a fluorescent dye 2', 7'-dichlorofluorescein diacetate (H_2DCF-DA, Sigma-Aldrich), as previously described (Zhou et al., 2016). Briefly, cells were washed with PBS and incubated with 10μM H_2DCF-DA at 37°C for 30 min to load the fluorescent dye. Cells were washed twice with PBS to remove unloaded fluorescent dye and trypsinized. Fluorescence (Ex.488 nm, Em.525 nm) was monitored by a Spectra-Max M5 Microplate Reader (Molecular Devices).

Immunofluorescence analysis

Mouse pancreases were fixed overnight in 4% paraformaldehyde at 4 °C, followed by paraffin embedding. Sections were deparaffinized, re-hydrated and incubated with mouse insulin antibody (Abcam, 1:1000) followed by detection with fluorescein-conjugated anti-rabbit.

Live Cell Imaging

The detailed live cell imaging experiment is described in previous study (Tucey et al., 2018). Briefly, BMDMs were seeded in cell culture plates at a density of 5×10^5 cells/well for 24-well plate and incubated overnight at 37°C with 5% CO_2. Macrophages were stained with 1 μM Cell Tracker Green CMFDA dye (Thermo Fisher C7025) for 20 min in serum-free DMEM. After one hour of co-incubated the

macrophages with one multiplicities of infection (MOI) of the *S. typhimurium* in low glucose (5mM glucose) or high glucose medium, non-phagocytosed bacterial were removed by washing three time with PBS; the cells were stained with 0.6 μM DRAQ7 (US Everbright Inc) in the corresponding medium and observed under a Leica AF6000 LX epifluorescence microscope for 24 h. The cell death data were analyzed and quantified using ImageJ.

RNA isolation and quantitative real-time PCR

Total RNA was isolated by Trizol according to manufacturer instructions. RNA was reverse-transcribed with oligo-dT primers and preceded to real-time PCR with gene-specific primers in the presence of SYBR Premix Ex Taq (Takara). Target gene expression was normalized to the housekeeping gene and relative quantitation values were calculated using the ΔΔ-CT method.

ELISA

Supernatant or serum was collected and ELISA performed according to manufacturers' instructions. For testing insulin levels, add 75 μl of Conjugate to each well, add 5 μl of each standard, control, and sample into their respective wells, then incubate for 2 h at room temperature with shaking at 700-900 rpm. After washing the microplate 6 times, add 100 μl of TMB Substrate into each well and incubate for 15 min at room temperature with shaking at 700-900 rpm. Finally, add 100 μl of Stop Solution and read at 450 nm within 30 min. For testing IL-1β levels, wash microwell strips twice with Wash Buffer. Add 100 μl of each standard, blank and sample into their respective wells, add 50 μl Biotin-Conjugate to all wells, then incubate for 2 h at room temperature. After

washing the microplate 4 times, add 100 μl Streptavidin-HRP to all wells and incubate 60 min at room temperature. After washing the microplate 4 times again, add 100 μl of TMB Substrate Solution into all wells and incubate for 10 min at room temperature. Finally, add 100 μl of Stop Solution and read at 450 nm within 30 min.

Western blotting

Protein concentration was quantified by the BCA kit (Thermo Fisher Scientific) and then subjected to SDS-PAGE and membrane transfer. The transferred membrane was blocked with 5% no-fat milk for 1 h at room temperature, and then primary antibody was incubated at 4°C overnight. Secondary antibody was incubated at room temperature for 1 h, and finally enhanced chemi luminescence (ECL) auto-development was performed.

Islet preparation and insulin secretion assay

Pancreatic islets were isolated from 8- to 12-week-old wild-type and *Sirt5*$^{-/-}$ mice by collagenase digestion and density-gradient centrifugation. Isolated islets were cultured with indicated reagents in RPMI 1640 medium (0.25% bovine serum albumin).

For insulin secretion assay, freshly isolated and incubated islets were washed twice with glucose-free phosphate buffer and were pre-incubated in Krebs-Ringer Buffer (KRB) containing 3.3mM glucose for 30 min. Then, ten islets per assay in triplicate were incubated with KRB buffer containing either 3.3mM glucose, 16.7mM glucose, or other reagents as indicated (LPS, 100ng/ml or IL-1β, 2.5ng/ml) for 1 h at 37°C. Supernatants containing insulin were removed and stored at − 20 °C until analysis. Insulin content was extracted with acid-ethanol. Insulin levels of all samples were measured by ELISA

kit.

Bone Marrow Transplantation

C57BL/6 recipient mice (6-week-old) were provided double-distilled drinking water with streptomycin (100μg/ml) and penicillin (100U/ml) for 7 days before radiation exposure. The recipient mice received whole body irradiation (γ-ray, 8Gy irradiation dose). Three hours later, each mouse was infused with 5×10^6 bone marrow cells of *Sirt5*$^{-/-}$ mice and WT mice via the angular vein, and finally formed *Sirt5*$^{-/-}$→WT and WT→WT groups. Experiments were initially conducted at 6 weeks after bone marrow reconstitution.

Statistics

All data shown represent the results obtained from two or three independent experiments with mean ± SEM. Statistical analyses were performed with a two-tailed unpaired Student's t-test. The values of $P < 0.05$ were considered statistically significant.

Key regents

REAGENT OR RESOURCE	SOURCE	IDENTIFIER
**Antibodies**		
Anti-Mouse/Rat IL-1β Functional grade purified	eBioscience	Cat#16-7012
Armenian Hamster IgG Isotype control Functional Grade purified	eBioscience	Cat#16-4888-85
Mouse IL-1β antibody	R&D	Cat#AF401-NA
Mouse β-actin antibody	Genescript	Cat#A00702
Mouse caspase-1 antibody	AdipoGen	Cat#AG-20B-0042
Phospho-Akt (Ser473) antibody	Cell Signaling Technology	Cat#4060
Akt (pan) antibody	Cell Signaling Technology	Cat#4685
Mouse CD11b PB antibody	eBioscience	Cat#48-0112-80
Mouse F4/80 FITC antibody	eBioscience	Cat#11-4801-81
Mouse insulin antibody	abcam	Cat#ab181547
**Chemicals and Recombinant proteins**		
Escherichia coli LPS 0111:B4	Sigma-Aldrich	Cat#L4391
Clodronate Liposomes	LIPOSOMA	NA
Control Liposomes(PBS)	LIPOSOMA	NA
ATP	Sigma-Aldrich	Cat#A6419
DPI(NAPDH oxidase inhibitor)	Alexis Biochemicals	Cat#BML-CN240
NAC(ROS inhibitor)	Sigma-Aldrich	Cat#A7250
Murine M-CSF	Peprotech	Cat#315-02
Novolin (human insulin)	Novo nordisk	-
H_2DCF-DA	Sigma-Aldrich	D6883
CMFDA dye	Thermo Fisher	C7025
DRAQ7	US Everbright Inc	D4076
**Critical Commercial Assays**		
Mouse IL-1β ELISA kit	eBioscience	BMS6002
Mouse Insulin ELISA kit	ALPCO	80-INSMSU-E01
SYBR® Premix Ex Taq™ (TliRNaseHPlus)	Takara	Cat #RR420A
PrimeScript™ RT master mix	Takara	Cat #RR036A
**Experimental Models: Organisms/Strains**		
Mouse: B6;129-Sirt5 tm1Fwa/J	The Jackson Laboratory	Cat #012757
Salmonella typhimurium (SL1344)		
**Primers**		

Mouse *Il-1β*<br>Forward: 5’-ACTGTTTCTAATGCCTTCCC-3’<br>Reverse: 5’-CGCAGCTCTAGGAGCATGTG-3’		
Mouse *Nlrp3*<br>Forward: 5’- ATTACCCGCCCGAGAAAGG -3’<br>Reverse: 5’- TCGCAGCAAAGATCCACACAG -3’		
Mouse *Actb*<br>Forward:5’-GGCTGTATTCCCCTCCATCG-3’<br>Reverse:5’-CCAGTTGGTAACAATGCCATGT-3’		

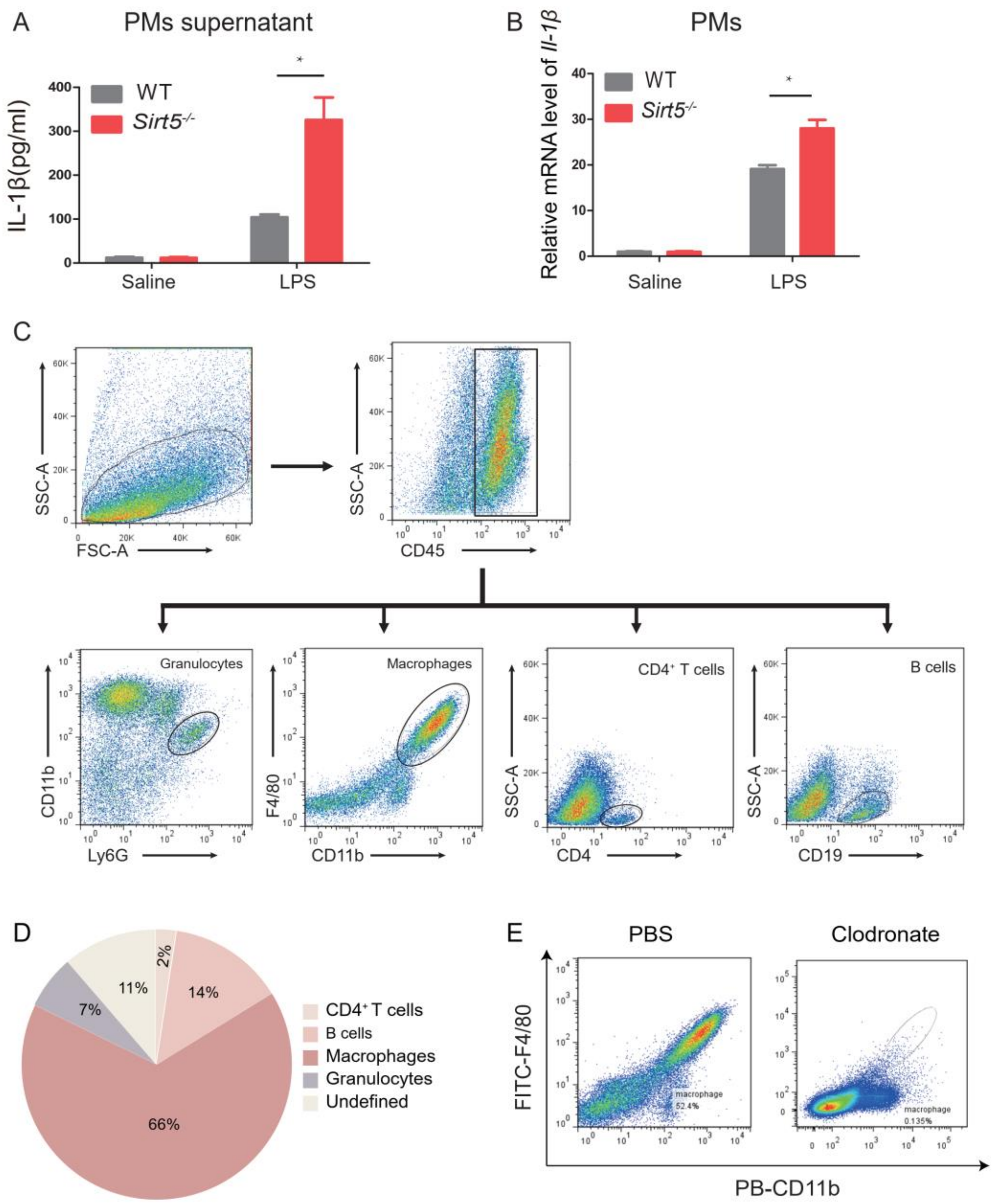

Figure S1 LPS stimulations IL-1β production in peritoneal macrophages from *Sirt5*$^{-/-}$ mice

(A) ELISA for the IL-1β released into the culture media of peritoneal macrophages (PMs) isolated from WT and *Sirt5*$^{-/-}$ mice (n=3) after intraperitoneal injection of LPS.

(B) The *Il-1β* mRNA level detected by qRT-PCR of PMs isolated from WT and *Sirt5*$^{-/-}$ mice (n=3) after intraperitoneal injection of LPS.

(C) FACS gating strategy for the analysis of mouse peritoneal cells.

(D) Peritoneal cell composition of *Sirt5*$^{-/-}$ mice with LPS intraperitoneal injection, determined by flow cytometry.

(E) Representative FACS plot of peritoneal macrophage (CD11b^{+}, F4/80^{+}) depletion in mice 3 days after injection of 10 ml/kg clodronate or PBS liposomes.

Data represent mean ± SEM, *p<0.05.

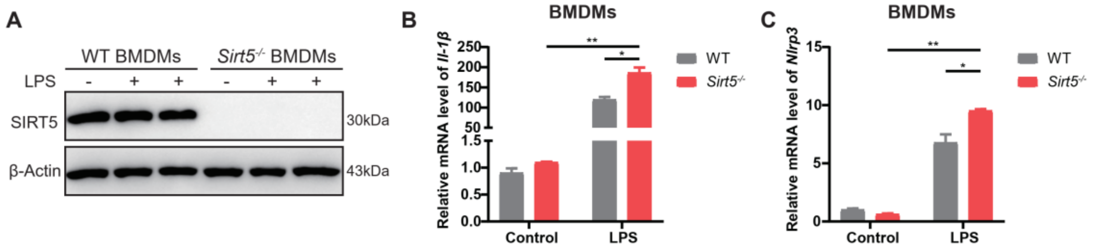

Figure S2. The mRNA level of *Il-1β* and *nlrp3* of BMDMs with LPS stimulation

(A) Immunoblot analysis of SIRT5 expression of BMDMs.

(B-C) The *Il-1β* (B) and *nlrp3* (C) mRNA level detected by qRT-PCR of BMDMs from WT and *Sirt5*$^{-/-}$ mice (n=3) after LPS stimulation.

Data represent mean ± SEM, *p<0.05, **p<0.01.

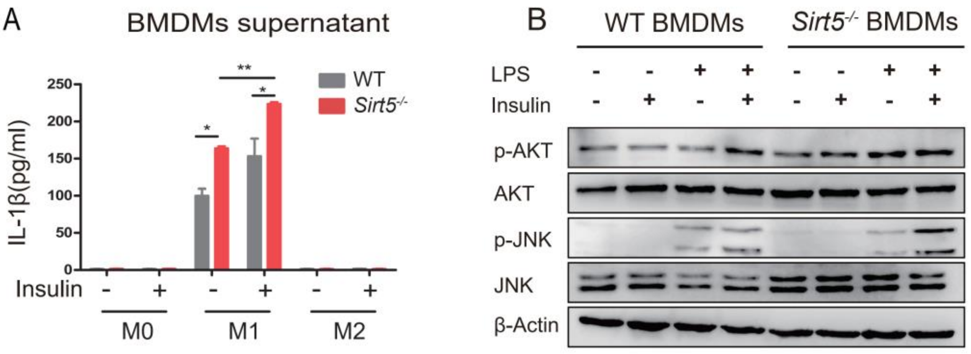

Figure S3. Insulin stimulates IL-1β secretion by macrophages

(A) Quantification of IL-1β by ELISA in the supernatant of M0, M1 and M2 macrophages treated for 5 min with insulin (1μg/ml) or not.

(B) Immunoblot analysis of AKT phosphorylated at Ser473 and phosphorylated JNK in BMDMs stimulated with or without LPS (100ng/ml, 3 h) and insulin (1μg/ml, 5 min). Data represent mean ± SEM, *$p<0.05$, **$p<0.01$.

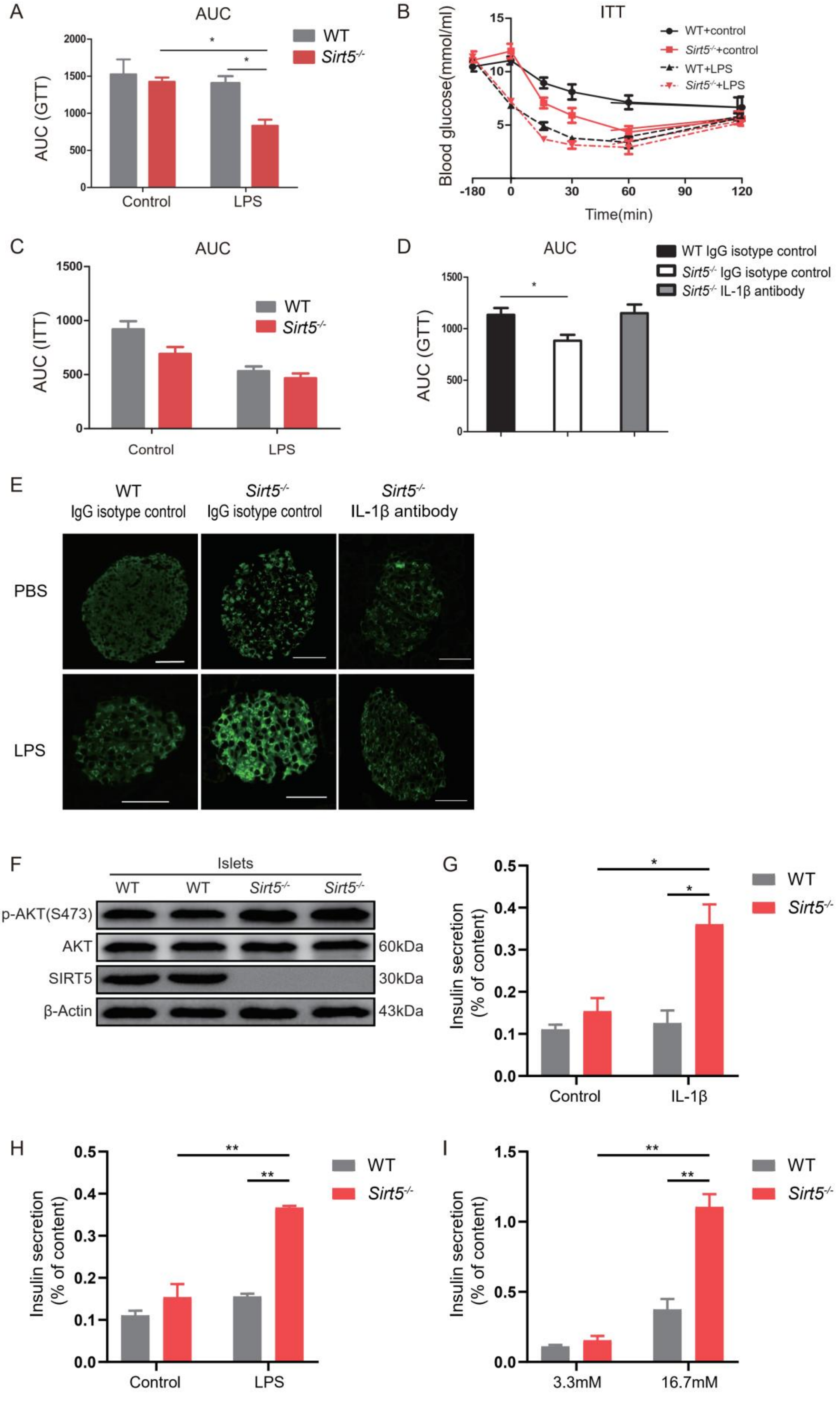

A
AUC
AUC (GTT)
WT
Sirt5-/-
Control
LPS
B
ITT
Blood glucose(mmol/ml)
Time(min)
WT+control
Sirt5-/-+control
WT+LPS
Sirt5-/-+LPS
C
AUC
AUC (ITT)
D
AUC
AUC (GTT)
WT IgG isotype control
Sirt5-/- IgG isotype control
Sirt5-/- IL-1β antibody
E
WT IgG isotype control
Sirt5-/- IgG isotype control
Sirt5-/- IL-1β antibody
PBS
LPS
F
Islets
p-AKT(S473)
AKT
SIRT5
β-Actin
60kDa
30kDa
43kDa
G
Insulin secretion (% of content)
Control
IL-1β
H
I
3.3mM
16.7mM

Figure S4 LPS stimulation induces insulin secretion due to increased IL-1β in *Sirt5*$^{-/-}$ mice

(A) The area under curve (AUC) during intraperitoneal GTT in WT or *Sirt5*$^{-/-}$ mice (corresponded to Figure 1M).

(B-C) Concentration of blood glucose (B) and the AUC (C) during intraperitoneal ITT in WT or *Sirt5*$^{-/-}$ mice after injection of saline (control) or LPS (1mg/kg) for 3 h (n=3/group); 0 min indicates ITT start time.

(D) The area under curve (AUC) during intraperitoneal GTT in WT or *Sirt5*$^{-/-}$ mice (corresponded to Figure 1O).

(E) Immunostaining of insulin (green) in pancreatic tissue sections of WT or *Sirt5*$^{-/-}$ mice 180 min after injection of PBS or LPS treated with IgG isotype control (100μg each) or IL-1β neutralizing antibody (100μg each). Scale bars=50μm.

(F) Immunoblot analysis of p-AKT(S473), AKT and SIRT5 protein levels in islets isolated from WT or *Sirt5*$^{-/-}$ mice.

(G) Islets isolated from WT or *Sirt5*$^{-/-}$ mice were stimulated with or without IL-1β (2.5ng/ml) at 3.3 mM glucose for 1 h and insulin secretion was assayed (n = 3).

(H) Islets isolated from WT or *Sirt5*$^{-/-}$ mice were stimulated with LPS (100ng/ml) glucose for 1 h, and insulin secretion was assayed (n = 3).

(I) Islets isolated from WT or *Sirt5*$^{-/-}$ mice were stimulated with 3.3 or 16.7 mM glucose for 1 h, and insulin secretion was assayed (n = 3).

Data represent mean ± SEM, *p<0.05, **p<0.01.

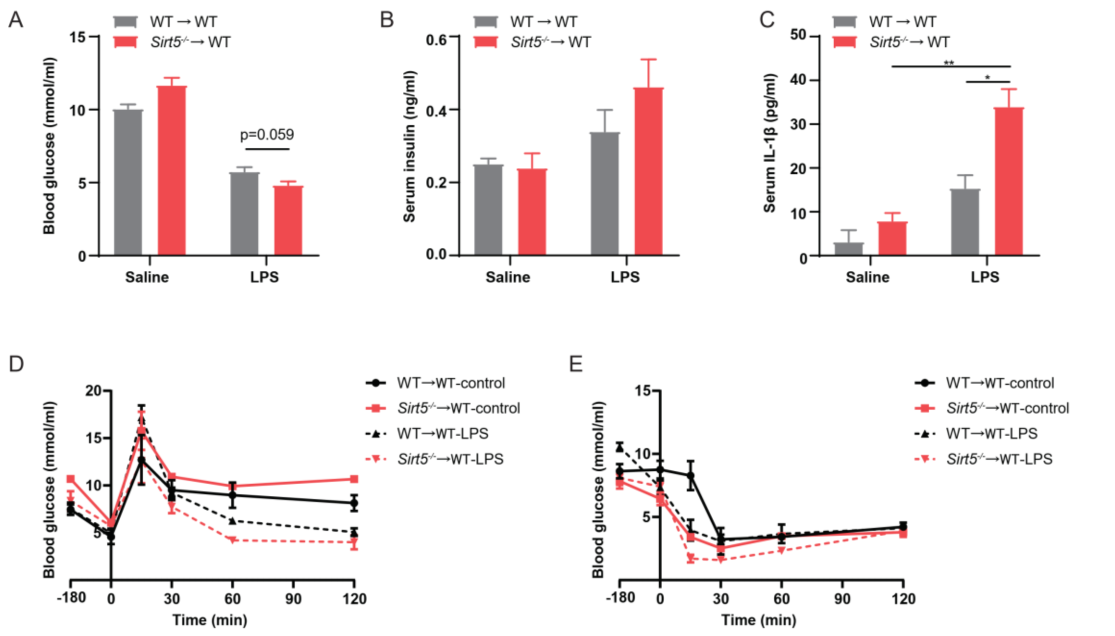

Figure S5 *Sirt5* deficiency in macrophages induced higher level of serum IL-1β

(A) Blood glucose, (B)serum insulin and (C) circulating IL-1β were determined before and after intraperitoneal injection of LPS for 3h (1mg/kg) into *Sirt5*$^{-/-}$→WT and WT→WT mice (n=3-4/group).

(D-E) Concentration of blood glucose during an intraperitoneal GTT and ITT analysis in *Sirt5*$^{-/-}$→WT and WT→WT mice after injection of saline (control) or LPS (1mg/kg) for 3 h (n=3-5/group).

Data represent mean ± SEM, $*p<0.05$, $**p<0.01$.

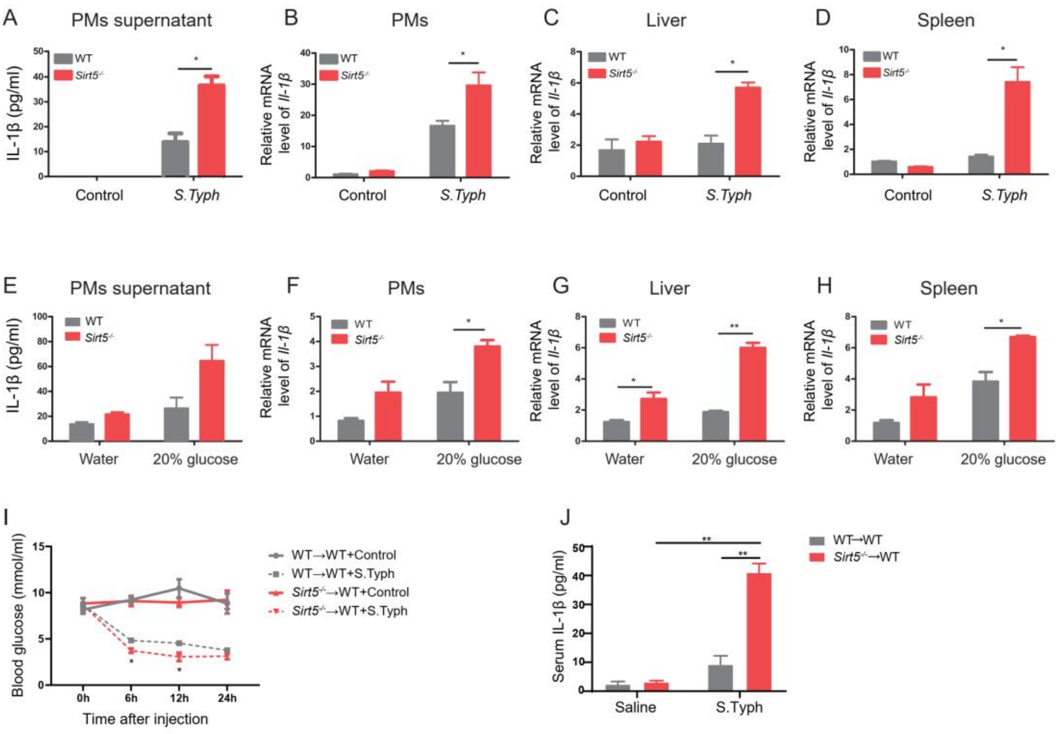

Figure S6 *Sirt5* deficiency stimulates of IL-1β production in *Salmonella typhimurium* infection model

(A-B) Mice were infected with *S. typhimurium* SL1344 strain (1 x 10^6 CFU/mouse), and sacrificed 24 h post infection. IL-1β in the supernatant of PMs (A) were measured by ELISA, the *Il-1β* mRNA level in PMs (B) was measured by qRT-PCR.

(C-D) Livers and spleens were extracted and the *Il-1β* mRNA level was measured by qRT-PCR.

(E-F) Mice were infected with *S. typhimurium* SL1344 strain (1 x 10^6 CFU/mouse) with or without 20% glucose in the drinking water, and sacrificed 24 h post infection. IL-1β in the supernatant of PMs (E) were measured by ELISA, the *Il-1β* mRNA level in PMs (F) was measured by qRT-PCR.

(G-H) Livers and spleens were extracted after 24 h infection and the *Il-1β* mRNA level was measured by qRT-PCR.

(I) Concentration of blood glucose of *Sirt5*$^{-/-}$→WT and WT→WT mice after a single intraperitoneal injection of *S. typhimurium* SL1344 strain (1 x 10^6 CFU/mouse) for 6 h, 12 h and 24 h.

(J) Mice were infected as in (I), and sacrificed 24 h post infection. IL-1β in serum was measured by ELISA.

Data represent mean ± SEM, *p<0.05, **p<0.01.

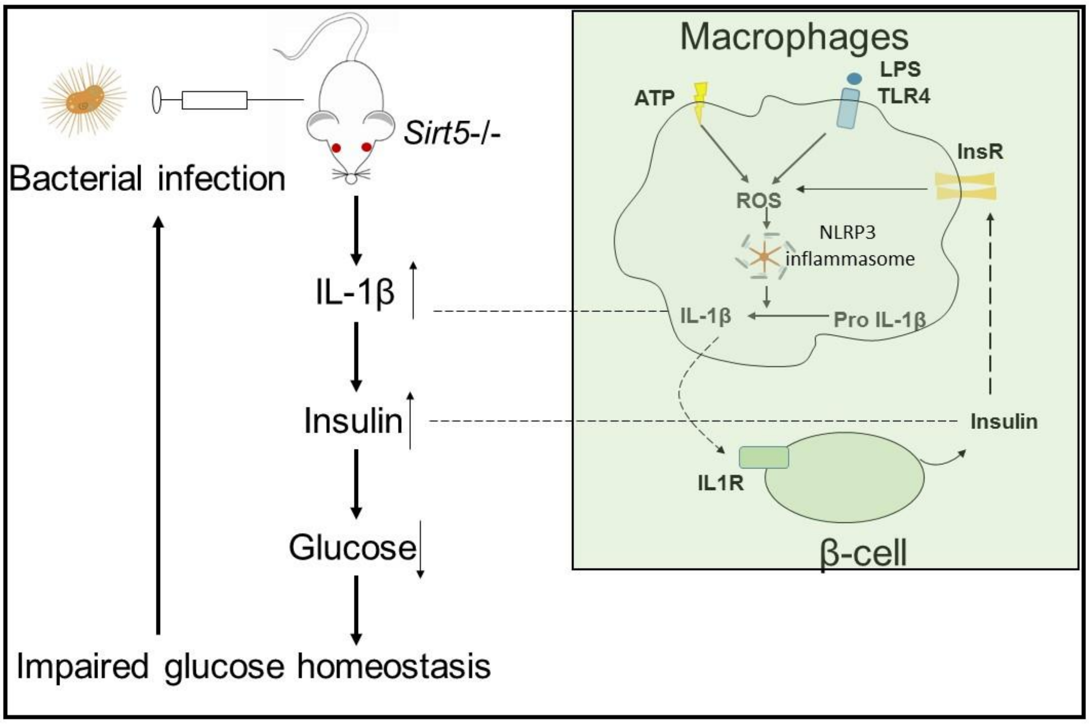

Figure S7 Working model of this work

We propose that there is a crosstalk between macrophage and pancreatic β cells , macrophage derived IL-1β and pancreatic β cells secreted insulin synergistically regulate glucose homeostasis. The peritoneal macrophages are stimulated by bacterial products to increase the production and release of IL-1β in *Sirt5*-/- mice. Increased IL-1β concentrations will then enhance insulin secretion from pancreatic β cells. The

secreted insulin binds to its receptor (InsR) on macrophages. This further stimulates macrophage- derived pro-IL-1β-maturation by the NLRP3 inflammasome. Finally, increased levels of IL-1β and insulin stimulate decrease glycemia, which is important for anti-systemic bacterial infection.

Reference

Tucey, T.M., Verma, J., Harrison, P.F., Snelgrove, S.L., Lo, T.L., Scherer, A.K., Barugahare, A.A., Powell, D.R., Wheeler, R.T., Hickey, M.J., et al. (2018). Glucose Homeostasis Is Important for Immune Cell Viability during Candida Challenge and Host Survival of Systemic Fungal Infection. Cell Metab 27, 988-1006 e1007.

Zhou, L., Wang, F., Sun, R., Chen, X., Zhang, M., Xu, Q., Wang, Y., Wang, S., Xiong, Y., Guan, K.L., et al. (2016). SIRT5 promotes IDH2 desuccinylation and G6PD deglutarylation to enhance cellular antioxidant defense. EMBO Rep 17, 811-822.
